# Beyond Smiles: Static Expressions in Maxillary Protrusion and Associated Positivity

**DOI:** 10.3389/fpsyg.2021.514016

**Published:** 2021-03-30

**Authors:** Lijing Chen, Jiuhui Jiang, Xingshan Li, Jinfeng Ding, Kevin B. Paterson, Li-Lin Rao

**Affiliations:** ^1^School of Psychology, Fujian Normal University, Fuzhou, China; ^2^CAS Key Laboratory of Behavioral Science, Institute of Psychology, Chinese Academy of Sciences, Beijing, China; ^3^School of Stomatology, Peking University, Beijing, China; ^4^Department of Neuroscience, Psychology and Behaviour, University of Leicester, Leicester, United Kingdom

**Keywords:** Implicit Association Test, implicit attitude, mandibular protrusion, smile, social-function account, maxillary protrusion

## Abstract

Smiles play an important role in social perception. However, it is unclear whether a similar role is played by static facial features associated with smiles (e.g., stretched mouth and visible teeth). In dental science, maxillary dental protrusions increase the baring of the teeth and thus produce partial facial features of a smile even when the individual is not choosing to smile, whereas mandibular dental protrusions do not. We conducted three experiments to assess whether individuals ascribe positive evaluations to these facial features, which are not genuine emotional expressions. In Experiment 1, participants viewed facial photographs of maxillary and mandibular protrusions and indicated the smiling and emotional status of the faces. The results showed that, while no difference was observed in participants’ perception of the presence of a smile across both types of dental protrusion, participants felt more positive to faces with maxillary than mandibular protrusions. In Experiment 2, participants completed an Implicit Association Test (IAT) test measuring implicit attitudes toward faces with maxillary vs. mandibular protrusions. The results showed that participants had more positive attitude toward faces with maxillary than mandibular protrusions. In Experiment 3, individuals with either maxillary or mandibular protrusions completed the same IAT test to assess whether any preference would be affected by in-group/out-group preferences. The results showed both groups had more positive attitudes toward faces with maxillary protrusion, indicating that this preference is independent of the group effect. These findings suggest that facial features associated with smiles are viewed positively in social situations. We discuss this in terms of the social-function account.

## Introduction

People often fabricate smiles in everyday lives (Ekman et al., 1988). Previous studies show that these can be distinguished from spontaneous smiles by differences in muscle patterns involved in the production of facial expressions (Ekman and Friesen, 1982; Ekman et al., 1988; Ekman, 1993; Kanade et al., 2000; Schmidt et al., 2006; Krumhuber and Manstead, 2009; Hossain et al., 2020). In a spontaneous smile, the zygomatic major pulls the mouth corner upwards and the orbicularis oculi raises the cheek and so produces certain features, which can include a raised upper lip, stretched mouth, and displayed teeth. By comparison, while fabricated smiles can share these features (Sabri, 2005), the orbicularis oculi usually does not move and the zygomatic major moves less far (Ekman and Friesen, 1982; Ekman et al., 1990; Krumhuber and Kappas, 2005; Guo et al., 2018). Considering that fabricated smiles are commonly used in daily interactions, it is possible that these play a similar role to spontaneous smiles, which may result from their common features. (see, e.g., Schmidt and Cohn, 2001; Mehu et al., 2007; Centorrino et al., 2015).

To date, this issue has been addressed by examining the social function of fabricated smiles. For example, Martin et al. (2017) recently proposed a social-functional account for smiles and defined three distinct smile expressions: reward smiles, affiliation smiles, and dominance smiles. Affiliation smiles, in particular, serve to establish and maintain mutual positive social bonds by signaling a friendly approach. More broadly, a behavioral ecology approach to understanding the function of facial expressions (Fridlund, 1994; Crivelli and Fridlund, 2018) suggests that these are social tools used to influence others. From this perspective, a smile may underpin a social intention (see also Schmidt and Cohn, 2001; Mehu et al., 2007; Centorrino et al., 2015). Substantial evidence also indicates that some expressions are more favored than others (Roelofs et al., 2009, 2010; Stins et al., 2011). For example, a happy face is socially more popular than an angry face. With the present research, we take a different approach to addressing this issue by examing the social perception of static facial features which are associated with smiles but also produced by a commonly occurring type of dental malocclusion, i.e., maxillary protrusion. These facial features include visible teeth and a stretched mouth, which are not produced by another type of dental malocclusion called mandibular protrusion.

**Figure 1:**
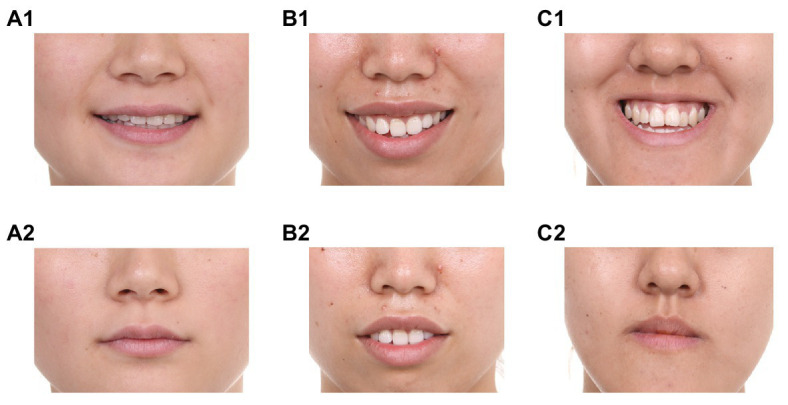
Examples of various occlusions. **(A1)** Normal occlusion with a posed smile; **(A2)** Normal occlusion with a neutral expression; **(B1)** Maxillary protrusion with a posed smile; **(B2)** Maxillary protrusion with a neutral expression; **(C1)** Mandibular protrusion with a posed smile; and **(C2)** Mandibular protrusion with a neutral expression.

These types of dental malocclusion are observed when the arrangement of the teeth deviates from normal occlusion (Olsen and Inglehart, 2011). In normal occlusion, the maxillary teeth cover the mandibular teeth by about 2 mm (Figures 1A1,A2). *If the maxillary teeth protrude further from the mandibular teeth* (> = *4 mm*; Figures 1B1,B2), **maxillary protrusion** is observed. Maxillary protrusion (called Type II protrusion in dental science) affects one in four people in the general population (Angle, 1907; Thilander et al., 2001; Borzabadi-Farahani et al., 2009). It increases the baring of the teeth, which is characteristic of a smile (Schmidt and Cohn, 2001). By comparison, *if the mandibular teeth cover the maxillary teeth* (Figures 1C1,C2), **mandibular protrusion** is observed (Type III protrusion in dental science), affecting one in 20 people (Angle, 1907; Thilander et al., 2001; Borzabadi-Farahani et al., 2009). This causes the jaw to protrude and does not create characteristics of a smile. Despite these differences in the effects on facial characteristics, maxillary and mandibular protrusions produce similar defects in chewing function, oral health and dental aesthetics (Angle, 1907; Olsen and Inglehart, 2011). Thus, facial features associated with a smile may be created by maxillary protrusion but not mandibular protrusion. A critical question, therefore, is whether smile-like facial features associated with maxillary protrusion, as opposed to mandibular protrusion, elicit the same social perceptions as smiles.

As we are focusing on the social perception of facial features associated with smiles, this raises the further issue of whether this perception is affected by the social groups to which individuals belong. Specifically, individuals with maxillary protrusion and those with mandibular protrusion may belong to different groups because they share different characteristics. Previous studies suggest that individuals generally have more positive attitudes toward other members of their own group (in-group) than those belonging to other groups (out-group), and so show in-group favoritism (Tajfel, 1970, 2010; Tajfel et al., 1971). Thus, according to in-group favoritism, individuals with maxillary protrusion may prefer maxillary protrusion, whereas those with mandibular protrusion prefer mandibular protrusion. On the other hand, sometimes individuals have more positive attitudes toward out-group individuals than in-group individuals, and so show out-group favoritism (Jost et al., 2002; Brewer, 2007). Out-group favoritism often occurs when individuals belong to lower-status groups, suggesting that if someone identifies his/herself as a lower-value individual, he/she identifies individuals similar to his/herself as other lower-value individuals (Jost et al., 2002; Brewer, 2007). Because individuals with malocclusions often seek orthodontic treatment, it is possible they are dissatisfied with their malocclusions. Thus, according to out-group favoritism, individuals with maxillary protrusion may dislike maxillary protrusion whereas individuals with mandibular protrusion dislike mandibular protrusion.

To test these hypotheses, we designed three experiments. In Experiment 1, we measured explicit perception of faces with maxillary and mandibular protrusions, including a cognitive measure that evaluates whether faces with maxillary and mandibular protrusions are perceived as if they are smiling, and an emotional measure that evaluates whether they are perceived as happy. In Experiment 2, we measured the implicit attitudes of participants with normal occlusion toward faces with maxillary and mandibular protrusions. Maxillary protrusion produces partial features of a smile whereas mandibular protrusion does not. If facial features of maxillary protrusion are viewed more positively than those of mandibular protrusion in social context, faces with maxillary protrusion may be more favored than those with mandibular protrusion; otherwise there should be no difference between the two. In Experiment 3, the same procedure was adopted to assess implicit attitudes toward the faces by participants with maxillary protrusion and those with mandibular protrusion. If participants’ attitudes are influenced by in-group favoritism, participants with maxillary protrusion may prefer faces with maxillary protrusion over faces with mandibular protrusion, whereas those with mandibular protrusion may show the opposite preference. However, if attitudes are influenced by out-group favoritism, those with maxillary protrusion may prefer faces with mandibular protrusion over maxillary protrusion, whereas those with mandibular protrusion may prefer faces with maxillary protrusion to mandibular protrusion. Moreover, if the presence of facial features associated with smiles affect implicit social perception independently of in-group and out-group favoritisms, both groups of participants might prefer faces with maxillary protrusion over those with mandibular protrusion.

Our findings will be valuable in extending understanding of the social perception of facial expressions and, in particular, how perceivers respond to smile-like expressions. Such findings will be relevant to the development of social-functional (Martin et al., 2017) and behavioral ecology (Fridlund, 1994; Crivelli and Fridlund, 2018) approaches to understanding the function of facial expressions, and especially smiles, in human societies. They may also contribute to the development of computational models of smile perception, by helping to understand how human observers respond to real and frabricated smiles, which in turn may support the optimization of artificial intelligence approaches to perceiving smiles in social interactions (Chen et al., 2018).

## Materials and Methods

### Ethics Approval Statement

The experiments were performed in accordance with approved guideline and regulations. All experimental protocols were approved by the Ethics Committee of the Peking University Stomatological Hospital. Written informed consent was obtained from all subjects. Consent to publish was obtained from the owners of the facial photographs.

### Participants

Twenty-four participants (undergraduate students from Fujian Normal University, mean age = 20 years, range = 18–23 years, and 12 females) took part in Experiment 1. Another 24 participants with normal occlusion (undergraduate students from Fujian Normal University who had no prior knowledge of malocclusion and did not take part in Experiment 1, mean age = 21 years, range = 19–24 years, and all males) participated in Experiment 2. Forty-eight participants with malocclusion, including 24 with severe maxillary protrusion and 24 with severe mandibular protrusion (patients seeking treatment at Department of Orthodontics, School of Stomatology, Peking University, mean age = 25 years, range = 19–35 years, and all females), participated in Experiment 3. These sample sizes were based on previous studies (Faul et al., 2007). All participants were paid a small amount for their participation.

### Materials

We selected 16 photographs of faces with malocclusion from a photographic database at Department of Orthodontics, School of Stomatology, Peking University, which includes 1,289 sets of photographs in total. The selection excluded photographs of models under 18 years old (i.e., juveniles) and over 60 years old (i.e., the elderly), faces with the other types of dental problem (e.g., missing teeth, crowded teeth, open bite, and jaw deformity), and those with only very slight light protrusion. From the remaining photographs, faces with moderate/serious protrusion were selected, while equating the age, gender and facial attraction of the faces selected with maxillary and mandibular protrusion. The final selection comprised eight faces with maxillary protrusion and eight with mandibular protrusion, each with four females (all with a neutral expression) and four males (two with a neutral expression and two with a posed smile, see Supplementary Figure 1). The 12 faces with a neutral expression were used as experimental materials and the four faces with posed smiles used as filler materials. For the 12 neutral faces, the six with maxillary protrusion showed more visible teeth than the six with mandibular protrusion [Maxillary Protrusion vs. Mandibular Protrusion = 3 vs. 0, *t*(5) = 3.67, *p* = 0.01]. The degree of malocclusion shown in the photographs was first assessed independently by two researchers. This was then evaluated using a five-point scale (1 = not at all, 2 = a little, 3 = moderately, 4 = very much, and 5 = extremely/severe) by 20 participants with normal occlusion (undergraduate students from Fujian Normal University who did not take part in the experiments; mean age = 21 years, range = 20–23 years, and eight females). The results showed that the perceived degree of malocclusion did not differ for maxillary compared to mandibular protrusion [Maxillary Protrusion vs. Mandibular Protrusion = 3.15 vs. 3.03, *t*(19) = 1.00, *p* = 0.33].

Sixteen stimulus words, comprising eight positive words and eight negative words (Supplementary Table 2), were selected, modified and translated into Chinese from positive and negative word lists constructed by Greenwald et al. (1998) and Greenwald and Farnham (2000) for the word classification task conducted in Experiments 2 and 3.

### Procedure

**Figure 2:**
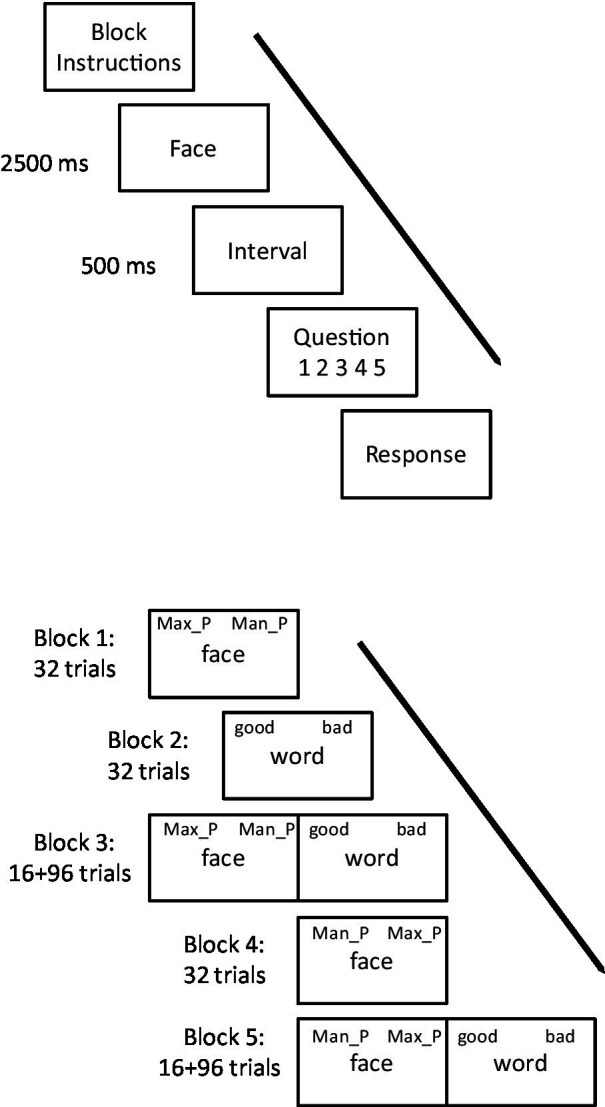
The experimental procedure for: **(A)** Experiment 1, where participants evaluate if faces are smiling in one block of trials, and happy in another block of trials, presented in counterbalanced order across participants; and **(B)** Experiments 2 and 3, using the Implicit Association Test (IAT) task. Max_P means maxillary protrusion; Man_P means mandibular protrusion. Participants responded in the IAT task using a computer keyboard, using the “f” and “j” keys to categorize face displays as Max_P vs. Man_P and word displays as “good” vs. “bad.” In Block 1, participants categorized faces only, in Block 2 they categorized words only, in Block 3 they categorized faces and words on 50% of trials each, in Block 4 they categorized faces only and, finally, in Block 5 they categorized faces and words on 50% of trials each. Half of the participants used the “f” key for Max_P responses in Blocks 1 and 3 and Man_P responses in Blocks 4 and 5, whereas the other half used this key for Man_P responses in Blocks 1 and 3 and for Max_P responses in Blocks 4 and 5. All participants used the “f” key for good responses in Blocks 2, 3, and 5.

In Experiment 1, participants were seated at a desk with a computer screen in a quiet room. The participants were instructed to evaluate a series facial photographs. Once they understood the task, the experiment began. The experiment included two blocks (Figure 2A). In one block, participants assessed whether the faces looked as if they were smiling or not; in the other block, the question asked about the emotional status of the faces. Half of the participants began with one block and the other half began with the second block. In each block, the photographs were displayed on the computer screen one by one. For each trial, the photograph was displayed for 2,500 ms. It then disappeared and, after a 500 ms interval, was replaced by the question (Supplementary Table 1). Participants answered the questions using five-point scales (for smile: 1 = looks serious, 5 = looks smiling; for emotional status: 1 = unhappy, 5 = happy). Each photograph was displayed once for the evaluation of smile and once for the evaluation of emotional status. For each participant, the order of presentation of photographs was randomized.

In Experiments 2 and 3, participants completed an Implicit Association Test (IAT; Greenwald et al., 1998) that measured their implicit attitudes toward the faces with maxillary/mandibular protrusion. An implicit attitude is an unconscious judgment that a thing is good or bad. These attitudes are activated automatically and can be measured by examining the speed of associating the target (i.e., maxillary protrusion vs. mandibular protrusion in the present study) with positive and negative attributes. In the present experiment, participants classified the 16 facial photographs as Maxillary Protrusion or Mandibular Protrusion by pressing one of two response keys. They also classified 16 adjectives as positive or negative in meaning by pressing the same response keys. Thus, a factor Combination was defined in terms of whether the positive words shared the same response key with photographs of maxillary protrusion (Maxillary-Positive Combination) or mandibular protrusion (Mandibular-Positive Combination). According to the standard IAT procedure, preferred photographs should be verified more quickly than dispreferred photographs when they share the same response key as positive words, whereas preferred photographs should be verified more slowly than dispreferred photographs when they share the same response key as negative words. In other words, *shorter* response time for *maxillary-protrusion* photographs in combination with positive words would indicate more *positive* attitude toward *maxillary protrusion*; whereas *shorter* response time for *mandibular-protrusion* photographs in combination with positive words would indicate more *positive* attitude toward *mandibular protrusion*.

At the beginning of the experiment, each participant read an expository text describing malocclusion and heard an experimenter’s introduction to the concepts of maxillary and mandibular protrusion. Once they indicated that they understood the concepts, the experiment began. Participants were then asked to classify the words as “good” or “bad” and the photographs as “maxillary protrusion” or “mandibular protrusion” as quickly and accurately as possible by pressing one of two response keys. The experiment contained five blocks, each lasting 2–3 min (Figure 2B). In the first block of trials, 16 facial photographs (half with maxillary protrusion and half with mandibular protrusion) were presented in the center of the screen one by one in random order. For half of the participants, the reminder label “maxillary protrusion” was located in the upper left corner, and “mandibular protrusion” in the upper right corner, of the screen. The participants had to classify maxillary-protrusion photographs as “maxillary protrusion” by pressing the left key and classify mandibular-protrusion photographs as “mandibular protrusion” by pressing the right key. The other half responded to mandibular-protrusion photographs by pressing the left key and responded to maxillary-protrusion photographs by pressing the right key, with the exchanged labels. Incorrect responses were indicated by a red cross displayed in the center of the screen; and participants had to correct the incorrect response. Each photograph appeared twice.

In the second block, the 16 photographs were replaced with 16 words (half positive, half negative). All the participants responded to positive words by pressing the left key and responded to negative words by pressing the right key, with the upper left label “good” and upper right label “bad.” In all other respects, this block was the same as the first block.

In the third block, the photograph classification task was inter-mixed with the word classification task. After 16 warm-up items (eight photographs and eight words), the 16 photographs and 16 words were mixed and displayed in a random order; each appearing three times. The response keys for photographs were inherited from the first block; and response keys for words inherited from the second block. Thus, half of the participants responded to maxillary-protrusion photographs and positive words using the same key; and the other half responded to mandibular-protrusion photographs and positive words using the same key.

In the fourth block, the first block was repeated, with the assignment of the response keys to the categories of photograph reversed, and the locations of the labels “maxillary protrusion” and “mandibular protrusion” exchanged. In the fifth block, the third block was repeated, with response keys for words inherited from the second block but response keys for photographs inherited from the fourth block. Thus the combinations of responses to photographs and words using the same keys were reversed.

### Data Analysis

Following standard statistical procedures, one-way ANOVA was adopted when there was one experimental factor whereas two-way ANOVA was adopted when there were two factors. Significance levels were set as *α* = 0.05. *Post hoc* power analyses are also reported (although we note the limitations of this approach; e.g., Lenth, 2007). In Experiment 1, a one-way repeated measure ANOVA was used to analyze responses to the 12 photographs of the faces with neutral expression for each question, treating Malocclusion Type (maxillary protrusion, mandibular protrusion) as the factor.

In Experiment 2, only response times for correct responses to 12 photographs with neutral expression in the third and fifth blocks were included in the analysis, excluding the eight warm-up items. The accuracy rate for responses was 0.95. There was no difference between the two types of Combination (maxillary protrusion + positive = 0.95, mandibular protrusion + positive = 0.95), *F* < 1. Response times more than 2.5 SDs from the mean were excluded as outliers. These procedures excluded 8.5% of the total data. Then a one-way repeated measure ANOVA was run for the remaining response time data, treating Combination (maxillary protrusion + positive, mandibular protrusion + positive) as the factor. The IAT effect was calculated by subtracting the response time in Maxillary-Positive Combination condition from that in Mandibular-Positive Combination condition. A *t* test was applied for the IAT effect.

Experiment 3 adopted the same procedure of preprocessing data. The accuracy rate for responses was 0.93. There was no difference across the four conditions (for participants with maxillary protrusion: maxillary protrusion + positive = 0.94, mandibular protrusion + positive = 0.93; for participants with mandibular protrusion: maxillary protrusion + positive = 0.94, mandibular protrusion + positive = 0.90), *F*s < 3.32. Incorrect responses and outliers were excluded from the analyses, accounting for 10.6% of the data. A two-way mixed measure ANOVAs were run with the remaining response time data, treating Group (participants with maxillary protrusion, participants with mandibular protrusion) and Combination (maxillary protrusion + positive, mandibular protrusion + positive) as factors. To examine the IAT effect for each participant group, the Experiment 2 analysis procedure was applied separately to results for participants with maxillary protrusion and those with mandibular protrusion.

## Results

### Experiment 1. Explicit Perception of Faces With Maxillary and Mandibular Protrusion

The analyses showed that there was no difference between maxillary protrusion and mandibular protrusion in relation to the perception of smile question [Maxillary Protrusion = 2.56, 95% CI 2.34–2.74, Mandibular Protrusion = 2.43, 95% CI 2.25–2.61, *F*(1, 23) = 1.16, *p* = 0.29, *η*^2^ = 0.05, observed power = 0.06]. However, on the evaluation of happiness, faces with maxillary protrusion received higher ratings than those with mandibular protrusion [Maxillary Protrusion = 2.87, 95% CI 2.74–3.00, Mandibular Protrusion = 2.53, 95% CI 2.32–2.73, *F*(1, 23) = 8.82, *p* = 0.01, *η*^2^ = 0.28, observed power = 0.48]. Thus, in the explicit evaluation, faces with maxillary protrusion were not reported as more smiling than faces with mandibular protrusion, suggesting that participants’ perception of a smile was not affected by malocclusion. However, the attribution of emotional status was affected by malocclusion, and faces with maxillary protrusion were perceived as happier than those with mandibular protrusion. This suggests that the facial characteristics of maxillary protrusion were taken as more positive than the facial characteristics of mandibular protrusion.

### Experiment 2. Implicit Attitudes Toward Maxillary Protrusion and Mandibular Protrusion

**Figure 3:**
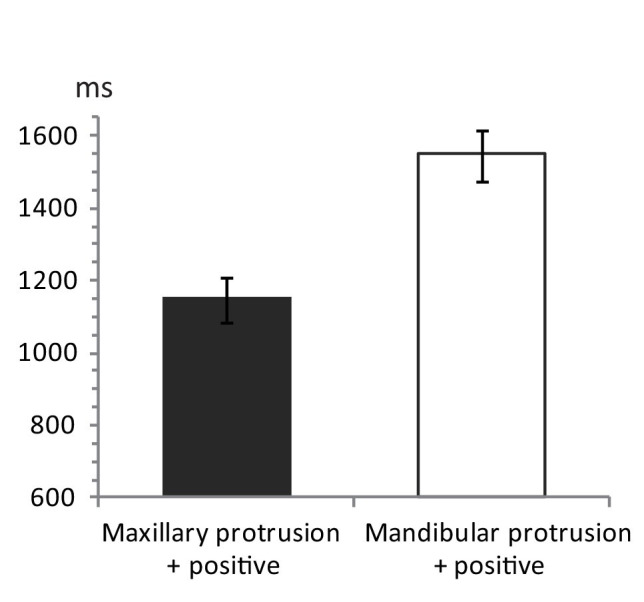
Mean response times in the IAT for 24 participants. Mean response time for the condition of maxillary-protrusion photographs + positive words (mandibular-protrusion + negative) is shown as dark gray bar, whereas for the condition of mandibular-protrusion photographs + positive words (maxillary protrusion + negative) is shown as white bar. The error bars indicate the SEM.

Mean response times to photographs in the two conditions are shown in Figure 3. An ANOVA comparing response times revealed a significant main effect of Combination, [*F*(1,23) = 25.19, *MSe* = 1,913,443, *p* < 0.001, *η*^2^ = 0.52, observed power = 0.94], due to participants responded more quickly to maxillary-protrusion photographs in combination with positive words (1,147 ms, 95% CI 1,023–1,271) than mandibular–protrusion photographs in combination with positive words (1,546 ms, 95% CI 1,398–1,694). The IAT effect (i.e., the difference that the response time in Mandibular-Positive Combination condition minus that in Maxillary-Positive Combination condition) was 399 ms (95% CI 235–564). A *t* test showed that this differed significantly from chance, [*t*(23) = 5.02, *p* < 0.001, Cohen’s *d* = 1.24, observed power = 1.0]. This indicates that individuals with normal occlusion have a more positive attitude toward maxillary protrusion than toward mandibular protrusion.

### Experiment 3. Implicit Attitudes of Individuals With Maxillary or Mandibular Protrusions

**Figure 4:**
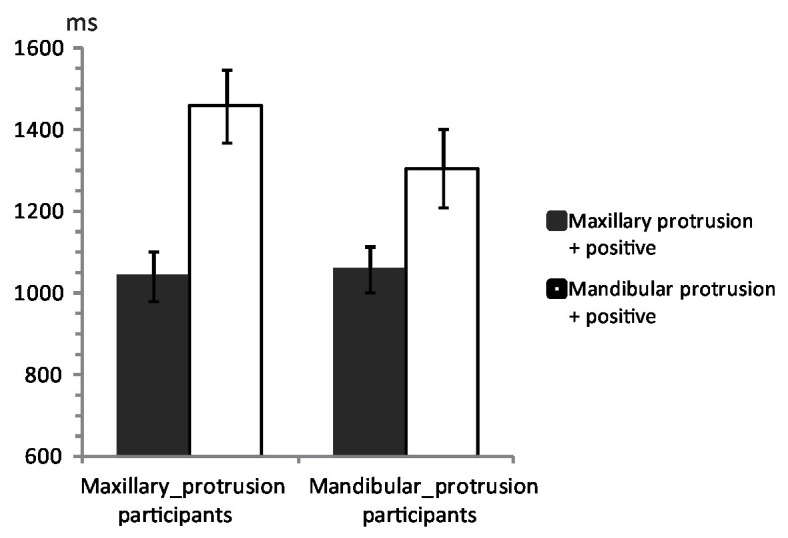
Mean response times in the IAT for 48 participants with malocclusion. Mean response times for the conditions of maxillary-protrusion photographs + positive words (mandibular-protrusion + negative) are shown as dark gray bars. Mean response times for the conditions of mandibular-protrusion photographs + positive words (maxillary-protrusion + negative) are shown as white bars. The left two bars show the results for the participants with maxillary protrusion, and the right two bars show the results for the participants with mandibular protrusion. The error bars indicate the SEM.

Mean response times to the photographs are shown in Figure 4. An ANOVA showed a significant main effect of Combinations, [*F*(1,46) = 33.15, *MSe* = 2,644,360, *p* < 0.001, *η*^2^ = 0.42, observed power = 0.94], such that photographs of maxillary protrusion in combination with positive words were identified more quickly (1,052 ms, 95% CI 970–1,134) than photographs of mandibular protrusion in combination with positive words (1,384 ms, 95% CI 1,253–1,515). No other effects were significant, *F*s < 2.20. The results indicate that both groups of participants had more positive attitudes toward maxillary protrusion than mandibular protrusion. To examine the IAT effect for the different groups of participants, a one-factor (Combinations) ANOVA was conducted separately for each group. The results showed that participants with maxillary protrusion responded more quickly to photographs of maxillary protrusion in combination with positive words (1,044 ms, 95% CI 920 to 1,167) than photographs of mandibular protrusion in combination with positive words [1,461 ms, 95% CI 1,276–1,646; *F*(1,23) = 33.85, *MSe* = 2,090,664, *p* < 0.001, *η*^2^ = 0.60, observed power = 0.98]. The IAT effect was 417 ms [95% CI 269–566; *t*(23) = 5.82, *p* < 0.001, Cohen’s *d* = 1.15, observed power = 1.0]. Participants with mandibular protrusion also responded more quickly to photographs of maxillary protrusion in combination with positive words (1,060 ms, 95% CI 944–1,177) than photographs of mandibular protrusion in combination with positive words [1,307 ms, 95% CI 1,112–1,502; *F*(1,23) = 7.45, *MSe* = 728,990, *p* = 0.01, *η*^2^ = 0.24, observed power = 0.37]. The IAT effect was 246 ms [95% CI 60–433; *t*(23) = 2.73, *p* = 0.01, Cohen’s *d* = 0.67, observed power = 0.88]. These results indicate that both groups had more positive attitudes toward maxillary than mandibular protrusion.

## General Discussion

The present study examined explicit evaluations and implicit attitudes toward faces with either maxillary or mandibular protrusion. There are several interesting findings. First, in Experiment 1, while participants reported no difference in the perception of a smile in neutral photographs of faces with maxillary and mandibular protrusion, they evaluated faces with maxillary protrusion as happier than those with mandibular protrusion. Second, in Experiment 2, participants without a dental occlusion had more positive implicit attitudes toward faces with maxillary than mandibular protrusion. Finally, in Experiment 3, individuals with either maxillary or mandibular protrusion had more positive implicit attitudes toward faces with maxillary than mandibular protrusion.

The emotional and attitudinal measures consistently showed that faces with maxillary protrusion are perceived as more positive and more favored than faces with mandibular protrusion. Facial features produced by maxillary protrusion (i.e., visible teeth and stretched mouth) are characteristics of a smile, even though the models in the photographs were not actually smiling. By comparison, mandibular protrusion does not produce facial features that are characteristic of a smile. Thus, the findings suggest that facial features associated with a smile are favored over those associated with non-smiles, similarly to the preference for spontaneous smiles over non-smiles (Roelofs et al., 2009, 2010; Stins et al., 2011). The results therefore suggest that static facial features associated with smiles can influence social perception. Moreover, this misattribution may occur even when the smile is not spontaneous, and may explain why pretending to smile can have positive outcomes in many social situations.

Another interesting result is that a preference for maxillary protrusion over mandibular protrusion was independent of in-group and out-group favoritisms. According to in-group favoritism (Tajfel, 1970, 2010; Tajfel et al., 1971), individuals with maxillary protrusion should prefer maxillary protrusion, whereas those with mandibular protrusion should prefer mandibular protrusion. On the other hand, according to out-group favoritism (Jost et al., 2002; Brewer, 2007), individuals with maxillary protrusion should dislike maxillary protrusion whereas those with mandibular protrusion should dislike mandibular protrusion. However, the results of Experiment 3 showed that both groups of participants favored photographs depicting maxillary protrusion over those depicting mandibular protrusion. These result are inconsistent with effects of in-group or out-group favoritism. However, they are consistent with the results of Experiment 2, revealing that individuals with either type of dental malocclusion exhibit the same preference for a malocclusion that displays static facial features associated with smiles. This suggests there is a reliable preference for the characteristics of a smile over a non-smile across different social groups.

These findings are clear. However, we must also consider some limitations to our approach. The first is that we assess the perception of smile-like facial expressions relative to different dental malocclusions only. Consequently, how these compare with genuine smiles is unclear. A valuable extension to the present work would therefore involve including photographs of spontaneous smiles as control stimuli and perhaps also to include these relative to fabricated (i.e., acted) smiles. In general, we consider that our approach may be valuable in the assessing the social perception of different types of fabricated smile. A further limitation to our approach relates to the use of real photos. This was intentional, as we used photos of real dental occlusions to maximize the ecological validity of the study. However, this also means that the stimuli comprised a variety of faces. Consequently, while we were careful to match faces in each dental occlusion condition in terms of sex, age and facial attractiveness, the stimuli may have different in other respects that might potentially have influenced the findings. The number of stimuli used was relatively small, as were the sample sizes of participants, and further work setting to replicate or extend the present findings would benefit from both increasing the stimulus set and the sample size to increase statistical power. Our sample sizes were also limited to patient and student groups and so further work is required to establish if the same effects are observed in the broader population. More positively, we note that the findings we obtained were as predicted with effect sizes of *η*^2^ = 0.24 and larger. This suggests that the effect sizes we investigated were of small to medium size or larger (e.g., Cohen, 1988) and that our methods were sufficient to detect these effects. The *post hoc* observed power for our experiments was also high for the majority of the effects we observed, suggesting that our study was sufficiently well powered to detect these effects.

Maxillary and mandibular protrusions deviate from normal occlusion to a similar physical degree (only in different directions), and affect chewing function, oral health and facial aesthetics similarly (Olsen and Inglehart, 2011). Therefore, the difference between the attitudes toward maxillary and mandibular protrusion are unlikely to be related to physical differences or differences in the symptoms experienced by individuals. It instead seems likely that they reflect differences in social perceptions of the characteristics of smiles and non-smiles. That is, because maxillary protrusion produces a characteristic of smile, others may interpret this facial feature as positive (Otta et al., 1996; Schmidt and Cohn, 2001; O’Doherty et al., 2003; Kaukomaa et al., 2013), and thus a positive attitude is formed. Moreover, such reactions may encourage positive social interactions (Tidd and Lockard, 1978; Scharlemann et al., 2001), consistent with implications of the social-functional account (Martin et al., 2017).

We consider that these findings contribute to our understanding of how smiles affect social intereaction and will be relevant to the development of social-functional (Martin et al., 2017) and behavioral ecology (Fridlund, 1994; Crivelli and Fridlund, 2018) approaches to understanding the function of facial expressions, and especially smiles, in human societies. We also note that the types of dental occlusion we examined are quite common (maxillary protrusions can be found in about 25% of the population, and mandibular protrusion in about 5% of the population; Angle, 1907; Thilander et al., 2001; Borzabadi-Farahani et al., 2009). This incidence may have implications for research on smiling, by highlighting the contribution of dental occlusion to an individual’s facial expressions. Such issues may also be important in the context of counseling individuals with different types of dental occlusion, by recognizing that this might affect their social interactions. Finally, this and other research might contribute to the development of computational models of smile perception, by helping to understand how human observers respond to real and fabricated smiles, which in turn may support the optimization of artificial intelligence approaches to perceiving smiles in social interactions (Chen et al., 2018).

In sum, the findings from the present experiments on perceptions of faces with dental malocclusion provide novel insights into how smiles, whether spontaneous or simulated, can facilitate positive social interaction. We also considered how these findings might relate to theories of social interaction and note that they may inform the development of artificial systems that can recognize and respond appropriately to the emotions communicated by human facial expressions.

## Data Availability Statement

The datasets generated for this study are available on request to the corresponding author.

## Ethics Statement

The experiments were performed in accordance with approved guideline and regulations. All experimental protocols were approved by the Ethics Committee of the Peking University Stomatological Hospital. Written informed consent was obtained from all subjects. Consent to publish was obtained from the owners of the facial photographs.

## Author’s Note

Maxillary protrusion is a dental malocclusion that causes the maxillary dental and/or jaw to protrude from the mandibular dental and/or jaw excessively, resulting in exposure of the upper teeth.

## Author Contributions

LC and JJ developed the research ideas and conducted the experiments. LC, JJ, XL, JD, and L-LR designed the experiments. LC analyzed the data and wrote the manuscript. LC, KP, XL, JD, L-LR, and JJ revised the manuscript. All authors contributed to the article and approved the submitted version.

### Conflict of Interest

The authors declare that the research was conducted in the absence of any commercial or financial relationships that could be construed as a potential conflict of interest.
