# Supplementary material for: Beyond Smiles: Static Expressions in Maxillary Protrusion and Associated Positivity
Source: Front Psychol. 2021 Mar 30;12:514016. doi: 10.3389/fpsyg.2021.514016 (PMC8042222; doi:10.3389/fpsyg.2021.514016)
Supplement: Supplementary file 1 [file Data_Sheet_1.pdf]

*Supplementary Material*

# 1 Supplementary Figure

A1

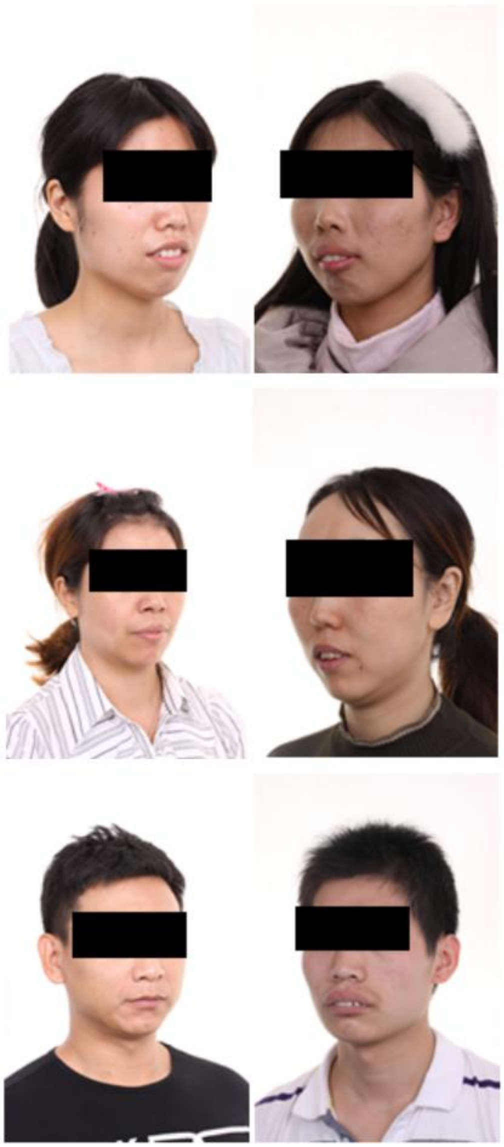

B1

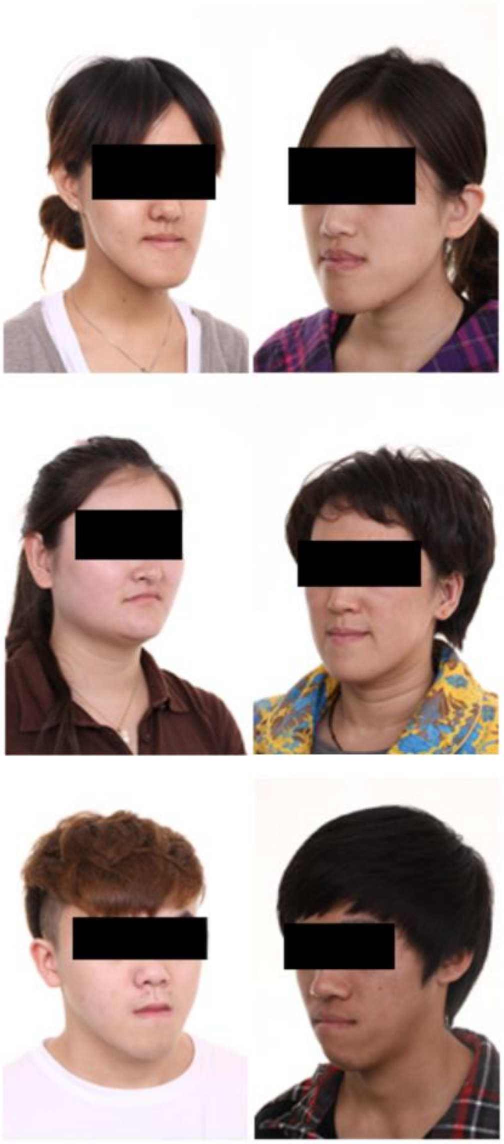

A2

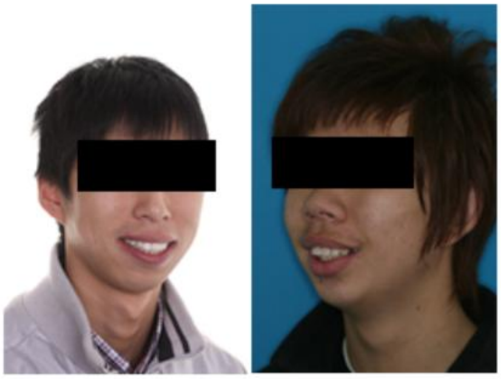

B2

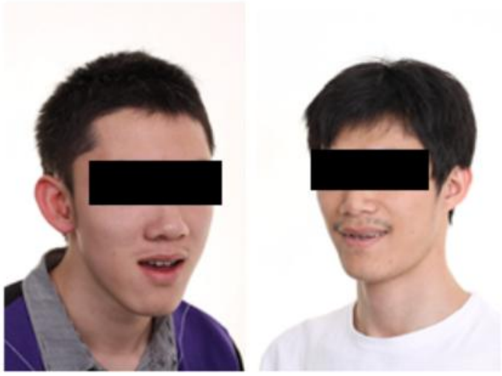

**Supplementary Figure 1.** (A1) 6 facial photographs with maxillary protrusion with a neutral expression; (A2) 2 facial photographs with maxillary protrusion with a posed smile; (B1) 6 facial photographs with mandibular protrusion with a neutral expression; (B2) 2 facial photographs with mandibular protrusion with a posed smile. The black bars on the facial photographs are presented to protect individuals' identities. They were not presented in the actual study. Participants saw the full and clear facial photographs in the actual experiments. The individuals in the photographs provided informed consent for use of their photographs for study and publication purposes.

## 2 Supplementary Tables

**Supplementary Table 1.** Questions used to evaluate the 12 facial photographs in Experiment 1

| | | | | | |
|---|---|---|---|---|---|
| 1、您觉得他/她看起来在笑吗？<br>Do you think it looks as if he/she is smiling? | 看起来严肃<br>Looks serious | | 中性<br>Neutral | | 看起来在笑<br>Looks smiling |
| | 1 | 2 | 3 | 4 | 5 |
| 2、您觉得他/她的情绪状态怎么样？<br>What do you think is his/her emotional status? | 不开心<br>Unhappy | | 中性<br>Neutral | | 开心<br>Happy |
| | 1 | 2 | 3 | 4 | 5 |

Note: This is a translation of the original questions in Chinese. In each trial, only one question was displayed. And for each question, only one of the pronouns (*he*, *she*, *his*, or *her*) was used as appropriate to the gender of the face in the photograph male.

**Supplementary Table 2.** The 16 stimulus words used in Experiment 2 and 3

| |
|---|
| Positive words:<br>喜悦、愉快、和平、欢笑、美妙、爱、光荣、快乐<br>Joyful, pleasant, peace, cheerful, wonderful, love, honor, happy |
| Negative words:<br>恐怖、伤害、可怕、邪恶、糟糕、肮脏、失败、苦恼<br>horror, hurt, terrible, evil, bad, dirty, failure, troubled |

Note: Only the Chinese versions of the words were presented in the experiments.
